# Supplementary material for: The gut microbiome mediates adaptation to scarce food in Coleoptera
Source: Environ Microbiome. 2023 Nov 13;18:80. doi: 10.1186/s40793-023-00537-2 (PMC10644639; doi:10.1186/s40793-023-00537-2)
Supplement: Supplementary file 3 — Supplementary Material 3 [file 40793_2023_537_MOESM3_ESM.qzv › 2edb1c1e-351e-4e50-8882-d2e663e43ad0/data/templates/standalone-template.html]

Emperor
{% include base\_dependencies\_path %}
{% include style\_template\_path %}

{% include html\_container\_path %}
