# Supplementary material for: The gut microbiome mediates adaptation to scarce food in Coleoptera
Source: Environ Microbiome. 2023 Nov 13;18:80. doi: 10.1186/s40793-023-00537-2 (PMC10644639; doi:10.1186/s40793-023-00537-2)
Supplement: Supplementary file 3 — Supplementary Material 3 [file 40793_2023_537_MOESM3_ESM.qzv › 2edb1c1e-351e-4e50-8882-d2e663e43ad0/data/templates/logic-template.html]

// When running in the Jupyter notebook we've encountered version conflicts
// with some dependencies. So instead of polluting the global require context,
// we define a new context.
var emperorRequire = require.config({
'context': 'emperor',
// the left side is the module name, and the right side is the path
// relative to the baseUrl attribute, do NOT include the .js extension
'paths': {
/\* jQuery \*/
'jquery': '{{ base\_url }}/vendor/js/jquery-2.1.4.min',
'jqueryui': '{{ base\_url }}/vendor/js/jquery-ui.min',
'jquery\_drag': '{{ base\_url }}/vendor/js/jquery.event.drag-2.2.min',
/\* jQuery plugins \*/
'chosen': '{{ base\_url }}/vendor/js/chosen.jquery.min',
'spectrum': '{{ base\_url }}/vendor/js/spectrum.min',
'position': '{{ base\_url }}/vendor/js/jquery.ui.position.min',
'contextmenu': '{{ base\_url }}/vendor/js/jquery.contextMenu.min',
/\* other libraries \*/
'underscore': '{{ base\_url }}/vendor/js/underscore-min',
'chroma': '{{ base\_url }}/vendor/js/chroma.min',
'filesaver': '{{ base\_url }}/vendor/js/FileSaver.min',
'blob': '{{ base\_url }}/vendor/js/Blob',
'canvastoblob': '{{ base\_url }}/vendor/js/canvas-toBlob',
'd3': '{{ base\_url }}/vendor/js/d3.min',
/\* THREE.js and plugins \*/
'three': '{{ base\_url }}/vendor/js/three.min',
'orbitcontrols': '{{ base\_url }}/vendor/js/three.js-plugins/OrbitControls',
'projector': '{{ base\_url }}/vendor/js/three.js-plugins/Projector',
'svgrenderer': '{{ base\_url }}/vendor/js/three.js-plugins/SVGRenderer',
'canvasrenderer': '{{ base\_url }}/vendor/js/three.js-plugins/CanvasRenderer',
'selectionbox': '{{ base\_url }}/vendor/js/three.js-plugins/SelectionBox',
'selectionhelper': '{{ base\_url }}/vendor/js/three.js-plugins/SelectionHelper',
/\* SlickGrid \*/
'slickcore': '{{ base\_url }}/vendor/js/slick.core.min',
'slickgrid': '{{ base\_url }}/vendor/js/slick.grid.min',
'slickformatters': '{{ base\_url }}/vendor/js/slick.editors.min',
'slickeditors': '{{ base\_url }}/vendor/js/slick.formatters.min',
'slickdataview': '{{ base\_url }}/vendor/js/slick.dataview.min',
/\* Emperor's objects \*/
'util': '{{ base\_url }}/js/util',
'model': '{{ base\_url }}/js/model',
'multi-model': '{{ base\_url }}/js/multi-model',
'view': '{{ base\_url }}/js/view',
'controller': '{{ base\_url }}/js/controller',
'draw': '{{ base\_url }}/js/draw',
'scene3d': '{{ base\_url }}/js/sceneplotview3d',
'shapes': '{{ base\_url }}/js/shapes',
'animationdirector': '{{ base\_url }}/js/animate',
'trajectory': '{{ base\_url }}/js/trajectory',
'uistate': '{{ base\_url }}/js/ui-state',
/\* controllers \*/
'abcviewcontroller': '{{ base\_url }}/js/abc-view-controller',
'viewcontroller': '{{ base\_url }}/js/view-controller',
'colorviewcontroller': '{{ base\_url }}/js/color-view-controller',
'visibilitycontroller': '{{ base\_url }}/js/visibility-controller',
'opacityviewcontroller': '{{ base\_url }}/js/opacity-view-controller',
'scaleviewcontroller': '{{ base\_url }}/js/scale-view-controller',
'shapecontroller': '{{ base\_url }}/js/shape-controller',
'axescontroller': '{{ base\_url }}/js/axes-controller',
'animationscontroller': '{{ base\_url }}/js/animations-controller',
/\* editors \*/
'shape-editor': '{{ base\_url }}/js/shape-editor',
'color-editor': '{{ base\_url }}/js/color-editor',
'scale-editor': '{{ base\_url }}/js/scale-editor'
},
/\*
Libraries that are not AMD compatible need shim to declare their
dependencies.
\*/
'shim': {
'jquery\_drag': {
'deps': ['jquery', 'jqueryui']
},
'chosen': {
'deps': ['jquery'],
'exports': 'jQuery.fn.chosen'
},
'contextmenu' : {
'deps': ['jquery', 'jqueryui', 'position']
},
'filesaver' : {
'deps': ['blob']
},
'canvastoblob' : {
'deps': ['blob']
},
'slickcore': ['jqueryui'],
'slickgrid': ['slickcore', 'jquery\_drag', 'slickformatters', 'slickeditors',
'slickdataview']
}
});
emperorRequire(
["jquery", "model", "controller"],
function($, model, EmperorController) {
var DecompositionModel = model.DecompositionModel;
var div = $('#{{ plot\_id }}');
var data = {{ data | tojson }};
var plot, biplot = null, ec;
function init() {
// Initialize the DecompositionModel for the scatter plot, and optionally
// add one for the biplot arrows
plot = new DecompositionModel(data.plot.decomposition,
data.plot.metadata\_headers,
data.plot.metadata,
data.plot.type);
if (data.biplot) {
biplot = new DecompositionModel(data.biplot.decomposition,
data.biplot.metadata\_headers,
data.biplot.metadata,
data.biplot.type);
}
ec = new EmperorController(plot, biplot, {{ plot\_id | tojson }}, undefined,
data.info);
}
function animate() {
requestAnimationFrame(animate);
ec.render();
}
$(window).resize(function() {
ec.resize(div.innerWidth(), div.innerHeight());
});
$(function(){
init();
animate();
ec.ready = function () {
// any other code that needs to be executed when emperor is loaded should
// go here
ec.loadConfig(data.plot.settings);
// sets up generic callbacks for 3rd party consumers
var plotView = ec.sceneViews[0];
/\*\_\_custom\_on\_ready\_code\_\_\*/
{{ js\_on\_ready }}
}
});
}); // END REQUIRE.JS block
