# Supplementary figures and images for: The gut microbiome mediates adaptation to scarce food in Coleoptera
Source: Environ Microbiome. 2023 Nov 13;18:80. doi: 10.1186/s40793-023-00537-2 (PMC10644639; doi:10.1186/s40793-023-00537-2)

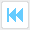

Supplement: Supplementary file 3 — Supplementary Material 3 [file 40793_2023_537_MOESM3_ESM.qzv › 2edb1c1e-351e-4e50-8882-d2e663e43ad0/data/img/reset.png]

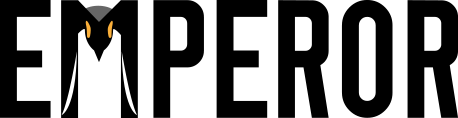

Supplement: Supplementary file 3 — Supplementary Material 3 [file 40793_2023_537_MOESM3_ESM.qzv › 2edb1c1e-351e-4e50-8882-d2e663e43ad0/data/img/emperor.png]

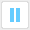

Supplement: Supplementary file 3 — Supplementary Material 3 [file 40793_2023_537_MOESM3_ESM.qzv › 2edb1c1e-351e-4e50-8882-d2e663e43ad0/data/img/pause.png]

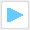

Supplement: Supplementary file 3 — Supplementary Material 3 [file 40793_2023_537_MOESM3_ESM.qzv › 2edb1c1e-351e-4e50-8882-d2e663e43ad0/data/img/play.png]

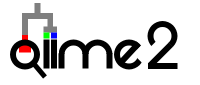

Supplement: Supplementary file 3 — Supplementary Material 3 [file 40793_2023_537_MOESM3_ESM.qzv › 2edb1c1e-351e-4e50-8882-d2e663e43ad0/data/q2templateassets/img/qiime2-rect-200.png]

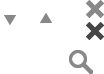

Supplement: Supplementary file 3 — Supplementary Material 3 [file 40793_2023_537_MOESM3_ESM.qzv › 2edb1c1e-351e-4e50-8882-d2e663e43ad0/data/vendor/css/chosen-sprite@2x.png]

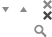

Supplement: Supplementary file 3 — Supplementary Material 3 [file 40793_2023_537_MOESM3_ESM.qzv › 2edb1c1e-351e-4e50-8882-d2e663e43ad0/data/vendor/css/chosen-sprite.png]

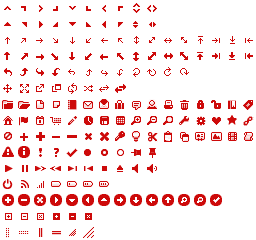

Supplement: Supplementary file 3 — Supplementary Material 3 [file 40793_2023_537_MOESM3_ESM.qzv › 2edb1c1e-351e-4e50-8882-d2e663e43ad0/data/vendor/css/images/ui-icons_cd0a0a_256x240.png]

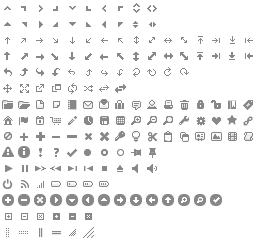

Supplement: Supplementary file 3 — Supplementary Material 3 [file 40793_2023_537_MOESM3_ESM.qzv › 2edb1c1e-351e-4e50-8882-d2e663e43ad0/data/vendor/css/images/ui-icons_888888_256x240.png]

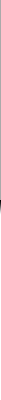

Supplement: Supplementary file 3 — Supplementary Material 3 [file 40793_2023_537_MOESM3_ESM.qzv › 2edb1c1e-351e-4e50-8882-d2e663e43ad0/data/vendor/css/images/ui-bg_glass_75_dadada_1x400.png]

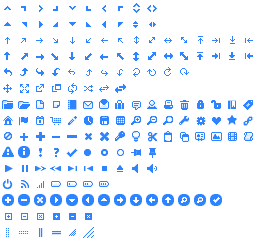

Supplement: Supplementary file 3 — Supplementary Material 3 [file 40793_2023_537_MOESM3_ESM.qzv › 2edb1c1e-351e-4e50-8882-d2e663e43ad0/data/vendor/css/images/ui-icons_2e83ff_256x240.png]

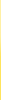

Supplement: Supplementary file 3 — Supplementary Material 3 [file 40793_2023_537_MOESM3_ESM.qzv › 2edb1c1e-351e-4e50-8882-d2e663e43ad0/data/vendor/css/images/ui-bg_highlight-soft_75_ffe45c_1x100.png]

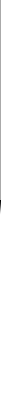

Supplement: Supplementary file 3 — Supplementary Material 3 [file 40793_2023_537_MOESM3_ESM.qzv › 2edb1c1e-351e-4e50-8882-d2e663e43ad0/data/vendor/css/images/ui-bg_glass_75_e6e6e6_1x400.png]

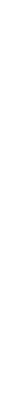

Supplement: Supplementary file 3 — Supplementary Material 3 [file 40793_2023_537_MOESM3_ESM.qzv › 2edb1c1e-351e-4e50-8882-d2e663e43ad0/data/vendor/css/images/ui-bg_glass_65_ffffff_1x400.png]

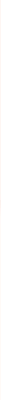

Supplement: Supplementary file 3 — Supplementary Material 3 [file 40793_2023_537_MOESM3_ESM.qzv › 2edb1c1e-351e-4e50-8882-d2e663e43ad0/data/vendor/css/images/ui-bg_glass_95_fef1ec_1x400.png]

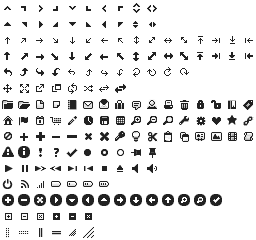

Supplement: Supplementary file 3 — Supplementary Material 3 [file 40793_2023_537_MOESM3_ESM.qzv › 2edb1c1e-351e-4e50-8882-d2e663e43ad0/data/vendor/css/images/ui-icons_222222_256x240.png]

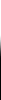

Supplement: Supplementary file 3 — Supplementary Material 3 [file 40793_2023_537_MOESM3_ESM.qzv › 2edb1c1e-351e-4e50-8882-d2e663e43ad0/data/vendor/css/images/ui-bg_highlight-soft_75_cccccc_1x100.png]

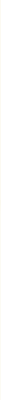

Supplement: Supplementary file 3 — Supplementary Material 3 [file 40793_2023_537_MOESM3_ESM.qzv › 2edb1c1e-351e-4e50-8882-d2e663e43ad0/data/vendor/css/images/ui-bg_glass_55_fbf9ee_1x400.png]

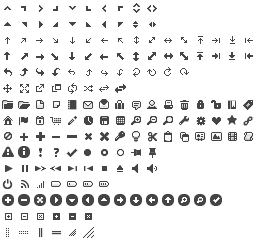

Supplement: Supplementary file 4 — Supplementary Material 4 [file 40793_2023_537_MOESM4_ESM.qzv › 701776f9-9920-4ea4-9e64-74319e4950ab/data/vendor/css/images/ui-icons_454545_256x240.png]
